# Anti-inflammatory diet consumption reduced fatty liver indices

**DOI:** 10.1038/s41598-021-98685-3

**Published:** 2021-11-19

**Authors:** Mitra Darbandi, Behrooz Hamzeh, Azad Ayenepour, Shahab Rezaeian, Farid Najafi, Ebrahim Shakiba, Yahya Pasdar

**Affiliations:** 1 Research Center for Environmental Determinants of Health (RCEDH), Health Institute, Kermanshah University of Medical Sciences, Kermanshah, Iran; 2 Social Development and Health Promotion Research Center, Kermanshah University of Medical Sciences, Kermanshah, Iran; 3 Nutritional Sciences Department, School of Nutritional Sciences and Food Technology, Kermanshah University of Medical Sciences, Isar Square, Kermanshah, Iran; 4 Cardiovascular Research Center, Kermanshah University of Medical Sciences, Kermanshah, Iran

**Keywords:** Diagnostic markers, Predictive markers

## Abstract

The aim of this study was to assess the association between dietary inflammatory index (DII) and non-invasive markers of liver status in adults. This cross-sectional study was performed on 8520 adults, recruited in Ravansar Non-Communicable Diseases (RaNCD) cohort study, western Iran. The DII score was calculated based on participants’ dietary intakes obtained from Food Frequency Questionnaire (FFQ). Fatty Liver Index (FLI) score was calculated by anthropometric measurements and some non-invasive markers of liver status. Linear regression models were applied to estimate the associations and adjust the possible confounding factors. A greater DII score was significantly associated with higher energy intake, body mass index (BMI), body fat mass (BFM), blood pressure, and FLI (P < 0.001). Participants with the highest DII score had a significantly higher consumption saturated fat, trans fat and red meat than those in the lowest quartile (P < 0.001). After adjustments of age and sex, participants in the highest quartile of the DII score had a greater risk of FLI (β: 0.742, 95% CI: 0.254, 0.601). More pro-inflammatory diet in participants was associated with a higher FLI. The DII score was positively associated with non-invasive liver markers. Thus, having an anti-inflammatory diet can help balance liver enzymes, reduce obesity, and decrease fatty liver.

## Introduction

Non-alcoholic fatty liver disease (NAFLD) is defined as the presence of ≥ 5% of hepatic steatosis without significant alcohol consumption^1^. NAFLD is the most common form of chronic liver disease worldwide that is the growing cause of end-stage liver disease. NAFLD recognized the contribution in the hepatocellular cancer (HCC) etiology^2^, and its prevalence is about 17–35% worldwide^3^. In recent decades, lifestyle changes including changes in habits and diet, decreased physical activity and increased the prevalence of obesity leading to a rise in NAFLD^2^. One third of the western populations has NAFLD^4^. Among the mentioned risk factors, one of the markers of NAFLD in the progressive stages is inflammation^5^. On the other hand, different studies reveal that diet can modulate inflammatory status^6^. Consequently, the evaluation of inflammatory potential of diet can be useful for the study of the association between diet and fatty liver. Employing different dietary indices is the common tool to study the association of diet quality and pathologic status including fatty liver disease^7^.

It has been proved that some diets such as western-like diets have a strong pro-inflammatory potential^8^. Dietary inflammatory index (DII) is a useful and interesting tool that has been developed and validated to evaluate the inflammatory potential of the overall diet^9^. Several studies have shown that DII is associated with diseases such as metabolic syndrome, cancer, and cardiovascular disease (CVD)^10–12^. A study by Cantero et al. implied that a pro-inflammatory diet may contribute to the development of fatty liver^13^.

Recently, a number of indices have been considered to predict fatty changes of the liver including the fatty liver index (FLI), hepatic steatosis index (HIS), NASH Score, and Steato test (ST)^14–16^. FLI developed by Bedogni et al. in 2006, is an equation comprising body mass index (BMI), waist circumference (WC), triglycerides (TG), and Gamma-Gluteamyl transferase (GGT) to predict fatty liver^14^. The equation is easy to use due to the fact that each of its components is a common clinical measurement, which has been validated as a practical, reliable, and economical tool for diagnosing NAFLD in large epidemiological studies^17^. Studies have indicated that FLI is an accurate alternative marker of hepatic steatosis in Asian and Western populations^18,19^. A study by Huang et al. (2015) examined the validity of FLI and revealed that it could accurately detect NAFLD with a good AUROC of 0.83 in the middle-aged and the elderly^20^ while Dehnavi et al. (2017) the AUC of FLI reported 0.85 in the diagnosis of NAFLD^21^. Therefore, the present work evaluated the associations of a validated DII, as a tool to assess the inflammatory capacity of the diet, with non-invasive liver markers in adults from the Ravansar Non-Communicable Diseases (RaNCD) cohort study.

## Results

### Characteristics of the participants

After applying the exclusion criteria among 10,063 participants, 8520 participants with a mean age of 47.24 ± 8.31 years were studied. Overall, 4275 (%50.18) were male, 1042 (%23.06) were current smokers, and 564 (%6.62) were alcohol consumers. The average of DII score was −2.32 ± 1.61 with a range of −6.18 to 4.27. In the study population, the average of FLI was 87.80 ± 3.99 with a range of 19.35 to 94.16.

Compared with those in the lowest quartile, participants in the highest quartile of DII score were younger (P < 0.001) and had a lower percentage of current smokers (Q1 = %23.06 vs. Q4 = %18.82, P < 0.001). A greater DII score was significantly associated with the consumption of alcohol (P < 0.001). There were significant differences in BMI, SBP, DBP, FLI, TG, BFM, ALT, and ALT/AST across quartiles of the DII score. No significant differences were found in WC and VFA across quartiles of the DII score (Table 1).

**Table 1:** Baseline characteristics, anthropometric and biochemical of participant’s factors according to the dietary inflammatory index score.

Variables	Total	Quartiles of dietary inflammatory index (DII)				P value*
		Q1	Q2	Q3	Q4	
Frequency	8520	2124	2089	2121	2186	–
DII score, mean ± SD	−2.32 ± 1.61	−4.04 ± 0.41	−3.13 ± 0.25	−2.10 ± 0.38	−0.08 ± 1.05	–
Age (year)	47.24 ± 8.31	48.85 ± 8.52	47.42 ± 8.34	46.59 ± 8.17	46.13 ± 7.98	< 0.001
Sex (% male)	4275 (50.18)	888 (20.77)	966 (22.60)	1098 (25.68)	1323 (30.95)	< 0.001
Current smoker, n (%)	1042 (23.06)	233 (30.14)	255 (27.04)	254 (21.01)	300 (18.82)	< 0.001
Use alcohol, n (%)	564 (6.62)	103 (4.85)	115 (5.51)	143 (6.74)	203 (9.29)	< 0.001
Weight (kg)	73.07 ± 13.67	70.10 ± 13.42	72.26 ± 13.27	73.77 ± 13.63	76.05 ± 13.67	< 0.001
BMI (kg/m^2^)	27.44 ± 4.60	26.97 ± 4.62	27.35 ± 4.60	27.53 ± 4.63	27.90 ± 4.54	< 0.001
BFM (kg)	33.47 ± 9.52	24.22 ± 9.22	24.90 ± 9.56	25.12 ± 9.66	25.26 ± 9.72	< 0.001
WHR	0.94 ± 0.06	0.93 ± 0.06	0.94 ± 0.06	0.94 ± 0.06	0.94 ± 0.06	< 0.001
WC (cm)	97.10 ± 10.51	97.10 ± 10.84	97.28 ± 10.34	97.25 ± 10.45	96.75 ± 10.37	0.328
VFA	120.92 ± 51.51	119.29 ± 50.70	121 ± 51.65	121.80 ± 51.86	121.08 ± 51.80	0.386
TG (mg/dl)	138.64 ± 85.58	135.40 ± 82.90	136.51 ± 79.61	138.33 ± 83.36	144.11 ± 95.06	0.004
AST (UI/L)	21.51 ± 8.88	21.34 ± 7.97	21.39 ± 8.40	21.40 ± 8.33	21.88 ± 10.52	0.161
ALT (UI/L)	25.04 ± 14.73	23.23 ± 13.18	24.21 ± 14.25	25.69 ± 15.31	26.95 ± 15.76	< 0.001
AST/ALT ratio	0.96 ± 0.34	1.02 ± 0.33	0.98 ± 0.38	0.93 ± 0.32	0.90 ± 0.28	< 0.001
GGT (UI/L)	361.40 ± 262.39	350.58 ± 261.90	360.99 ± 268.67	363.83 ± 63.16	369.92 ± 255.79	0.106
SBP (mm Hg)	108.50 ± 17.11	108.69 ± 18.10	107.25 ± 16.40	108.26 ± 17.06	109.74 ± 16.77	< 0.001
DBP (mm Hg)	70.03 ± 9.98	69.90 ± 10.21	69.20 ± 9.45	69.85 ± 9.95	71.13 ± 10.21	< 0.001
FLI	87.80 ± 2.86	87.45 ± 4.50	87.75 ± 3.98	87.90 ± 3.79	88.10 ± 3.62	< 0.001

*AST* aspartate transaminase, *ALT* alanine aminotransferase, *BFM* body fat mass,** BMI** body mass index, *DII* dietary inflammatory index, *FLI* fatty liver index, *GGT* gamma-glutamyl-transferase, *TG* triglycerides, *SBP* systolic blood pressure, *DBP* diastolic blood pressure, *VFA* visceral fat area, *WC* waist circumference, *WHR* waist hip ratio.

*Analysis of variance (ANOVA), P < 0.05.

### Food parameters participants according to the dietary inflammatory index score

A greater DII score was significantly associated with the higher intakes of energy (Q1 = 1935.18 ± 706.80 vs. Q4 = 2919.13 ± 1082.31; P < 0.001) resulting in a larger DII. and protein (Q1 = 14.93 ± 1.70 vs. Q4 = 16.59 ± 2.30; P < 0.001). The lower intakes of carbohydrate (Q1 = 66.32 ± 8.64 vs. Q4 = 62.62 ± 7.90; P < 0.001) resulted in a larger DII score. Participants with the highest DII had a significantly higher consumption saturated fat, PUFA, and MUFA than with those in the lowest quartile (P < 0.001) (Table2).

**Table 2:** Description of food parameters participants according to the dietary inflammatory index score.

Food parameters	Total	Q1	Q2	Q3	Q4	P value
Energy (kcal/day)	2328.53 ± 935.00	1935.18 ± 706.80	2081.70 ± 731.33	2356.85 ± 838.53	2919.13 ± 1082.31	< 0.001
Carbohydrate (%E)	64.57 ± 8.04	66.32 ± 8.64	65.53 ± 7.47	63.88 ± 7.55	62.62 ± 7.90	< 0.001
Protein (%E)	15.73 ± 2.05	14.93 ± 1.70	15.54 ± 1.89	15.81 ± 1.92	16.59 ± 2.30	< 0.001
Lipid (%E)	19.44 ± 7.65	18.10 ± 8.43	18.48 ± 7.10	20.11 ± 7.24	21.01 ± 7.38	< 0.001
Saturated fat (g/day)	20.38 ± 14.72	16.34 ± 12.92	16.83 ± 11.64	21.06 ± 13.54	27.03 ± 17.39	< 0.001
MUFA (g/day)	13.44 ± 12.26	9.89 ± 9.85	10.77 ± 9.29	13.91 ± 11.23	18.97 ± 15.39	< 0.001
PUFA (g/day)	4.10 ± 2.55	2.56 ± 1.44	3.27 ± 1.45	4.21 ± 1.88	6.26 ± 03.22	< 0.001
Trans fat (g/day)	0.06 ± 0.08	0.04 ± 0.05	0.05 ± 0.05	0.06 ± 0.06	0.10 ± 0.11	< 0.001
Cholesterol (mg/day)	259.11 ± 163.28	187.39 ± 20.15	219.57 ± 19.60	266.17 ± 41.24	359.73 ± 200.56	< 0.001
Red meat (g/day)	0.78 ± 1.15	0.66 ± 0.97	0.62 ± 0.92	0.81 ± 1.10	1.03 ± 1.47	< 0.001
Poultry (g/day)	1.58 ± 1.47	1.03 ± 0.87	1.37 ± 1.12	1.63 ± 1.41	2.28 ± 1.93	< 0.001
Fish (g/day)	0.19 ± 0.304	0.56 ± 0.15	0.13 ± 0.22	0.20 ± 0.28	0.32 ± 0.43	< 0.001
Vegetables (g/day)	4.86 ± 3.52	2.41 ± 1.47	3.61 ± 1.90	5.09 ± 2.40	8.23 ± 4.30	< 0.001
Fruits(g/day)	2.57 ± 2.22	1.41 ± 1.29	1.96 ± 1.46	2.77 ± 1.88	4.10 ± 2.86	< 0.001
Dairy product (g/day)	2.90 ± 2.25	2.42 ± 2.20	2.50 ± 1.95	3.02 ± 2.14	3.63 ± 2.48	< 0.001
Legumes (g/day)	2.86 ± 2.720	1.46 ± 1.14	2.12 ± 1.59	2.89 ± 2.13	4.90 ± 3.76	< 0.001
Egg (g/day)	0.37 ± 0.367	0.25 ± 0.28	0.33 ± 0.32	0.38 ± 0.34	0.50 ± 0.45	< 0.001
Potato (g/day)	0.52 ± 0.48	0.35 ± 0.30	0.46 ± 0.38	0.54 ± 0.43	0.74 ± 0.64	< 0.001
Refined grains (g/day)	5.59 ± 2.63	4.87 ± 1.90	5.25 ± 2.53	5.65 ± 2.49	6.55 ± 3.12	< 0.001
Whole grains (g/day)	0.75 ± 0.992	0.42 ± 0.55	0.57 ± 0.67	0.77 ± 0.92	1.24 ± 1.39	< 0.001
Nuts (g/day)	4.14 ± 6.66	1.91 ± 3.53	2.85 ± 4.32	4.36 ± 6.10	7.32 ± 9.51	< 0.001

### The association of fatty liver index with dietary inflammatory index

Crude and multivariable-adjusted linear regression analysis was used to investigate the effect of several factors on FLI (Table3). We found a significant association between the DII scores and FLI, with one-point increment in the DII score, on average the risk of having FLI increased by 13 (95% CI, 0.076 to 0.181). In the crude and multivariable-adjusted model with one-point increment in the physical activity, on average the risk of having FLI decreased by 6 (95% CI, − 0.076 to − 0.056) and 2 (95% CI, − 0.030, − 0.016), respectively. We also found a significant association between FLI and BMI, with one-point increment in the BMI, on average the risk of having FLI increased by 23 in the crude and 35 in the adjusted models.

**Table 3:** Association between selected variables and fatty liver index by **l**inear regression analysis.

FLI	Crude		Adjusted*	
	β (95% CI)	P value	β (95% CI)	P value
Age (year)	0.033 (0.023, 0.043)	< 0.001	0.028 (0.001, 0.58)	0.020
Sex	−0.082 (−0.252, 0.087)	0.111	−0.009 (−0.300, 0.067)	0.214
DII	0.130 (0.076, 0.181)	< 0.001	0.024 (−0.02, 0.073)	0.321
Energy intake (kcal day)	0.001 (0.006, 0.002)	0.002	0.0001 (0.000, 0.000)	0.050
BMI	0.232 (0.225, 0.239)	< 0.001	0.350 (0.330, 0.336)	< 0.001
BFM	0.232 (0.225, 0.240)	< 0.001	0.128 (0.122, 0.135)	< 0.001
WC	0.237 (0.231, 0.243)	< 0.001	0.124 (0.120, 0.131)	< 0.001
Physical Activity	−0.066 (−0.076, −0.056)	< 0.001	−0.023 (−0.030, −0.016)	< 0.001

*Adjusted for current smoker, use alcohol, FBS and lipid profile (LDL-C, HL-C, TG and total cholesterol).

**Figure 1:**
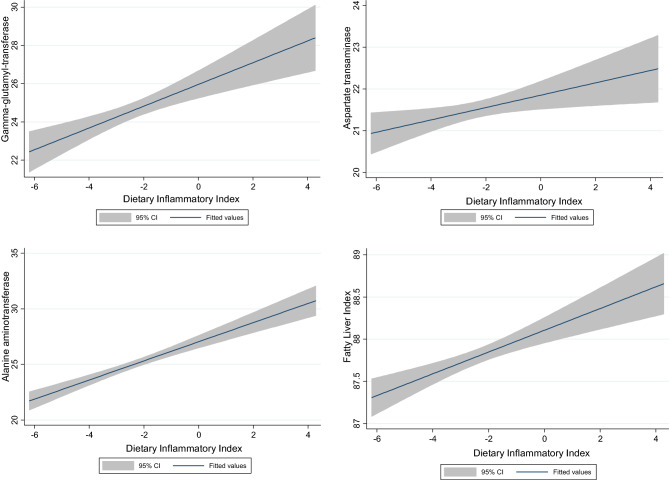
Associations between markers of liver status and dietary inflammatory index.

After adjustments of age and sex (Model 1, Table 4) and after adjustments of BMI, WC, and physical activity (Model 2, Table 4), participants in the highest quartile of the DII score had a greater risk of FLI (β: 0.742 and β: 0.497, respectively) than those in the lowest quartile. Figure 1 shows a positive association between DII and liver markers.

**Table 4:** Association between dietary inflammatory index and fatty liver index by **l**inear regression analysis.

	Quartiles of dietary inflammatory index (DII)						
	Q1	Q2 β (95% CI)	P value	Q3 β (95% CI)	P value	Q4 β (95% CI)	P value
Crude	Ref	0.293 (0.010, 0.336)	0.018	0.448 (0.032,0.3767)	< 0.001	0.643 (0.201, 0.542)	< 0.001
Model 1	Ref	0.345 (−0.023, 0.369)	0.005	0.532 (0.082, 0.428)	< 0.001	0.742 (0.254, 0.601)	< 0.001
Model 2	Ref	0.177 (−0.056, 0.219)	0.058	0.294 (−0.043, 0.232)	0.002	0.497 (0.116, 0.393)	< 0.001

Model 1: Adjusted for age and sex.

Model 2: Adjusted for age, sex, body mass index, waist circumference and physical activity.

## Discussion

Our findings showed that a higher pro-inflammatory diet in participants was associated with a higher FLI. The DII score was positively associated with the non-invasive liver markers (ALT, AST, and GGT), suggesting that diet-induced inflammation may increase liver disorders. We found a positive association between FLI and BMI, WC and BFM in crude and adjusted models. While this finding supports the idea that inflammation is induced by adiposity, the association may be bidirectional. In the study of Cantero et al.^13^, a positive association between the DII and FLI has also been observed, as it reveals that higher inflammation and fewer adherences to the Mediterranean diet were related with a higher degree of liver disorders in obese individuals.

In the current work, the level of inflammatory markers was not measured. However, given that the obesity is an inflammatory condition and the rise in BMI is associated with an increase in the DII score (Q1 = 26.97 vs. Q4 = 27.90), subsequently the association between DII and inflammatory conditions is inferred. A study by Fong et al. (2006) observed association between different diet-quality scores including the alternative healthy eating index (AHEI), healthy eating index (HEI), and the alternate Mediterranean diet index (aMED) with inflammatory markers such as CRP and IL-6^22^. Sureda et al. (2018) reported that low adherence to the Mediterranean dietary pattern (MDP) was directly associated with a worse profile of plasmatic inflammation markers including adiponectin, Leptin, TNF-α, plasminogen activator inhibitor 1 (PAI-1), and high-sensitivity C-reactive protein (hs-CRP)^23^.

The present study revealed that more pro-inflammatory diet in the participants was associated with higher energy intake, red meat, saturated fat, trans fat, cholesterol, MUFA, and PUFA. Previous studies have shown that healthy food is inversely related to inflammation^1,2^ and also several trial and meta-analysis studies have reported association between enough intake of vitamin C, vitamin E, antioxidants, and fiber with lower levels of inflammatory markers^3,4,6,7^. On the other hand, associations between DII and metabolic syndrome (MetS) components including high TG and low HDL-C concentrations, high blood pressure, glucose intolerance, central obesity, CVD, and liver cancer have been reported to be related^2,24–26^. Overall, the mentioned studies’ results are in line with our findings in the hypothesis that diet is an important factor in the inflammation process^13,24^.

In the crude and adjusted models with increasing physical activity, inflammation was reduced. A cohort study with a ten-year follow-up showed physically active participants at baseline had lower CRP and Il-6 levels, which remained stable over time^27^. There are other studies reporting an inverse association of physical activity with inflammatory markers^28–30^.

The liver has many functions within the body including amino acid synthesis, carbohydrate metabolism, cholesterol linkage, protein degradation, the manufacture of TG, and the major part of lipoprotein synthesis^31^. Currently, there is no effective treatment for NAFLD; therefore, many researches have recently focused on finding biomarkers for a prediction of liver disorders particularly NAFLD. FLI is a combination of four components: BMI, WC, TG, and GGT. Thus, calculating the FLIis simple, with an accurate and cost effective method^14^. According to the components of the FLI (including anthropometric measures, liver enzymes, and blood lipid), its increasing could be a type of inflammation and setting an anti-inflammatory diet can keep balance the FLI.

The main strength of our study is the large sample size. This study faced several limitations; firstly, not being able to evaluate the inflammatory markers. Therefore, we suggest that these markers be examined in future studies. Secondly, due to the cross-sectional nature of the study, it is not possible to infer causality and more studies are needed. In addition, because of the large sample size, more of the relationships were statistically significance which should not be over-emphasized if there is no clinical significance. Third, FFQ are containing some degree of recall bias. However, we used the validated dietary questionnaire for calculation of DII, thus diet was investigated through face-to-face interviews, which may have reduced measurement error. Although, the diet may change over time and we are unable to measure the changes; longitudinal studies evaluating these associations are needed to determine causality. Fourth, liver fat was not directly measured, i.e. magnetic resonance spectroscopy (MRS) or magnetic resonance imaging (MRI).

## Conclusion

Our study findings indicated that more pro-inflammatory diet in participants was associated with higher FLI. The DII score was also positively associated with non-invasive liver markers (ALT, AST, and GGT). We found a positive association between FLI with BMI, WC, BFM and energy intake, red meat, saturated fat, trans fat, cholesterol, MUFA, and PUFA. Therefore, it was concluded that having an anti-inflammatory diet can help balance liver enzymes, reduce obesity, and decline body fat mass and also related co-morbidities like the fatty liver.

## Methods

### Study population

**Figure 2:**
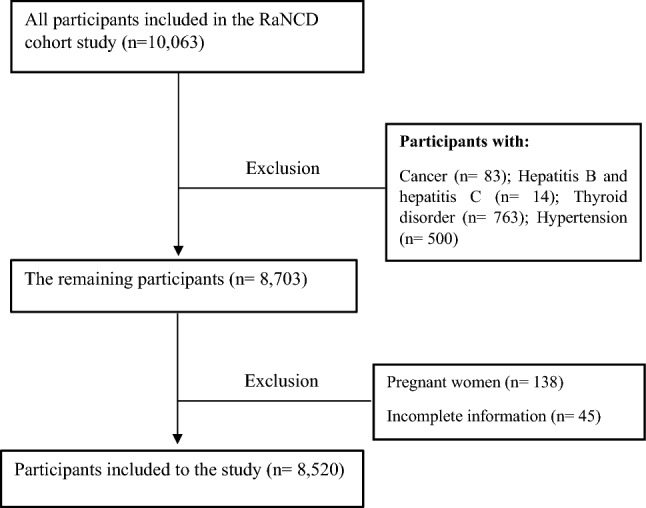
Flow chart of the study.

This was a cross-sectional study based on baseline data from Ravansar Non-Communicable Disease (RaNCD) prospective study in western Iran in 2020. The RaNCD study is part of a prospective epidemiological research study in Iran (PERSIAN). Ravansar is a district with urban and rural areas located in the west of Iran and in Kermanshah province with a population of about 50,000. The initial phase data was collected in 2014, and 10,000 adults between the ages of 35 and 65, being registered as permanent residents of Ravansar, were included in this cohort study. RaNCD study methodology and design with details have been published in 2019^32^. Participants included all subjects from the first phase of the RaNCD study. For this study, the exclusion criteria were as follows: pregnancy, patients with hepatitis B, hepatitis C, cancer, hypertension, thyroid disorder, and the case that their information was incomplete (Fig. 2).

### Ethics approval

The Ethics Committee of Kermanshah University of Medical Sciences approved the study (code: KUMS.REC.1399.640). All methods were carried out in accordance with relevant guidelines and regulations. All the participants were provided oral and written informed consent.

### Data collection

Data collection and all measurements of anthropometry and biochemical were conducted and assessed in the RaNCD cohort site. Participants were invited to the cohort center and the questionnaires were completed by trained experts. Demographic information and personal habits were completed face-to-face in the digital cohort questionnaire.

### Anthropometry measurements

We used the Bio-Impedance Analyzer BIA (Inbody 770, Inbody Co, Seoul, Korea) to measure body weight with a precision of 0.5 kg and BSM 370 (Biospace Co, Seoul, Korea) to measure height with the precision of 0.1 cm. Body composition components including BMI, Body fat mass (BFM), visceral fat area (VFA), Waist hip ratio (WHR), and WC were measured by BIA. WC also was measured with a flexible measuring tape at a level midway between the lowest rib margin and the iliac crest.

### Blood pressure

We measured blood pressure using a manometer cuff and stethoscope from both arm in the seated position and after ten minutes of rest for two times from each arm with an interval of five minutes. Then a mean of both systolic and diastolic blood pressure was reported.

### Biochemical factors

Serum and plasma samples were obtained according to standard protocol of RaNCD cohort study, after 8–12 h of fasting from the ante-brachial vein. Serum concentrations of liver enzymes including Alanine aminotransferase (ALT), Aspartate transaminase (AST), and GGT and lipid profiles including TG, LDL-C, HDL-C, and total cholesterol were measured by enzymatic kits (Pars Azmun, Iran), centrifugedand then stored in aliquots in cryotubes at –80 ◦C until the analysis.

### Fatty liver index

FLI was first introduced by Bedogni et al. in 2006 using the bootstrapped stepwise logistic regression analysis^14^, with thirteen variables (including gender, age, ethanol intake, ALT, AST, GGT, BMI, WC, sum of four skinfolds, glucose, insulin, TG, and cholesterol), four of which remained as predictors in the equation:

$$\mathrm{FLI}=\frac{({\mathrm{e}}^{ 0.953\times \mathrm{log}(\mathrm{e}) (\mathrm{TG}) + 0.139\times \mathrm{ BMI}+ 0.718\times \mathrm{ log }(\mathrm{e}) (\mathrm{GGT}) + 0.053 \times \mathrm{ WC }- 15.745}) }{(1+ {\mathrm{e}}^{ 0.953\times \mathrm{log}\left(\mathrm{e}\right)\left(\mathrm{TG}\right)+ 0.139\times \mathrm{ BMI}+ 0.718\times \mathrm{log}\left(\mathrm{e}\right)\left(\mathrm{GGT}\right)+ 0.053 \times \mathrm{ WC }- 15.745 }) }\times 100$$

The accuracy measured with area under the receiver operator characteristic curve (AUROC) of the FLI was 0.83 (95% CI: 0.825 to 0.842) in detecting fatty liver^20^. The FLI ranges from 0 to 100. Thus, FLI scores of < 30 and FLI ≥ 60 indicated the absence or presence, respectively, in fatty liver with a good diagnostic accuracy^14^.

### Assessment of DII

Dietary information derived from the Food Frequency Questionnaire (FFQ) was applied to calculate the DII scores for subjects. Shivappa et al. found that 45 food items were associated with one or more of the inflammatory including Interleukin-1b (IL-1b), Interleukin-6 (IL-6), Tumor Necrosis Factor-a (TNF-a), C-reactive protein (CRP), or anti-inflammatory markers including Interleukin-4 (IL-4) and Interleukin-10 (IL-10). They scored the inflammatory potential for each food parameter according to whether it increased inflammatory or decreased anti-inflammatory markers (+ 1), whether it decreased inflammatory or increased anti-inflammatory markers (−1), or if it had no effect (0) on the level of inflammatory or anti-inflammatory markers. They calculated global mean and standard deviation for each of the 45 food parameters based on 11 data sets from 11 countries in different parts of the world^9^.

In the present study, according to the food parameters in the Iranian questionnaire, we calculated the DII score based on 31 food parameters, foods, and nutrients that we were used in this study: vitamin A, vitamin B6, vitamin B12, vitamin C, vitamin D, vitamin E, folic acid, niacin, iron, zinc, selenium, magnesium, beta-carotene, caffeine, thiamin, riboflavin, onion, garlic, tea, omega-3, omega-6, trans fat, saturated fats (SFAs), cholesterol, mono-unsaturated fatty acids (MUFA), poly-unsaturated fatty acids (PUFA), fiber, protein, total fat, carbohydrate, and energy.

To calculate the DII score for each subject, we subtracted the "standard global average" from the value consumed per person and then divided by the "global standard deviation" to obtain the Z score for each food parameter. We used the global means ± SDs from the Shivappa et al. study^9^. Afterwards, we converted these values to a centered percentiles score to minimize the risk of skewness. The inflammatory score for each of the food parameters was calculated via this method, and then the inflammatory score of all parameters was summed to calculate the overall DII score which could be positive or negative. More positive DII scores indicate more pro-inflammatory diets, while more negative scores imply more inti-inflammatory diets^9,33^. Finally, DII scores were classified into four groups (quartile) to investigate the association between the different variables. The first and fourth quartiles had the lowest and highest DII scores, respectively.

### Statistical analysis

All analyses in this study were performed using Stata version 14.2 software (Stata Corp, College Station, TX, USA), which is available from: (https://downloadbull.com/portable-statacorp-stata-14-2-free-download/). General characteristics, anthropometric indices, and biochemical factors of participants across quartiles of the DII score were reported as mean ± standard deviation for continuous variables and as percentages for qualitative variables. The normality was checked using the Kolmogorov–Smirnov test. To compare differences across DII quartiles, we used the one-way ANOVA test. Analysis of linear regression was conducted to determine associations between FLI and the DII score and other risk factors adjusted for smoking and alcohol use. Additionally, linear regression was applied to determine the associations between FLI across quartiles of the DII score for adjusting the following confounding factors: age, sex, BMI, WC, and physical activity. Variables with p-value < 0.2 in univariable analysis were entered into multivariable linear model. For statistical analyses, a p-value of < 0.05 with 95% confidence intervals (CIs) was considered significant.

## Data Availability

The datasets generated and/or analyzed during the current study are not publicly available for ethical reasons, as well as privacy reasons but are available from the corresponding author on reasonable request.
